# In-vivo quantitative T_2_ mapping of carotid arteries in atherosclerotic patients: segmentation and T_2_ measurement of plaque components

**DOI:** 10.1186/1532-429X-15-69

**Published:** 2013-08-16

**Authors:** Luca Biasiolli, Alistair C Lindsay, Joshua T Chai, Robin P Choudhury, Matthew D Robson

**Affiliations:** 1 Oxford Centre for Clinical Magnetic Resonance Research (OCMR), Radcliffe Department of Medicine, University of Oxford, John Radcliffe Hospital, Oxford, UK; 2 Oxford Acute Vascular Imaging Centre (AVIC), Radcliffe Department of Medicine, University of Oxford, John Radcliffe Hospital, Oxford, UK

**Keywords:** Atherosclerosis, Cardiovascular magnetic resonance, Carotid plaque imaging, *In-vivo* T_2_ map, Plaque segmentation, Lipid-rich necrotic core, Fibrous tissue, AHA plaque type classification

## Abstract

**Background:**

Atherosclerotic plaques in carotid arteries can be characterized *in-vivo* by multicontrast cardiovascular magnetic resonance (CMR), which has been thoroughly validated with histology. However, the non-quantitative nature of multicontrast CMR and the need for extensive post-acquisition interpretation limit the widespread clinical application of *in-vivo* CMR plaque characterization. Quantitative T_2_ mapping is a promising alternative since it can provide absolute physical measurements of plaque components that can be standardized among different CMR systems and widely adopted in multi-centre studies. The purpose of this study was to investigate the use of *in-vivo* T_2_ mapping for atherosclerotic plaque characterization by performing American Heart Association (AHA) plaque type classification, segmenting carotid T_2_ maps and measuring *in-vivo* T_2_ values of plaque components.

**Methods:**

The carotid arteries of 15 atherosclerotic patients (11 males, 71 ± 10 years) were imaged at 3 T using the conventional multicontrast protocol and Multiple-Spin-Echo (Multi-SE). T_2_ maps of carotid arteries were generated by mono-exponential fitting to the series of images acquired by Multi-SE using nonlinear least-squares regression. Two reviewers independently classified carotid plaque types following the CMR-modified AHA scheme, one using multicontrast CMR and the other using T_2_ maps and time-of-flight (TOF) angiography. A semi-automated method based on Bayes classifiers segmented the T_2_ maps of carotid arteries into 4 classes: calcification, lipid-rich necrotic core (LRNC), fibrous tissue and recent IPH. Mean ± SD of the T_2_ values of voxels classified as LRNC, fibrous tissue and recent IPH were calculated.

**Results:**

In 37 images of carotid arteries from 15 patients, AHA plaque type classified by multicontrast CMR and by T_2_ maps (+ TOF) showed good agreement (76% of matching classifications and Cohen’s κ = 0.68). The T_2_ maps of 14 normal arteries were used to measure T_2_ of tunica intima and media (T_2_ = 54 ± 13 ms). From 11865 voxels in the T_2_ maps of 15 arteries with advanced atherosclerosis, 2394 voxels were classified by the segmentation algorithm as LRNC (T_2_ = 37 ± 5 ms) and 7511 voxels as fibrous tissue (T_2_ = 56 ± 9 ms); 192 voxels were identified as calcification and one recent IPH (236 voxels, T_2_ = 107 ± 25 ms) was detected on T_2_ maps and confirmed by multicontrast CMR.

**Conclusions:**

This carotid CMR study shows the potential of *in-vivo* T_2_ mapping for atherosclerotic plaque characterization. Agreement between AHA plaque types classified by T_2_ maps (+TOF) and by conventional multicontrast CMR was good, and T_2_ measured *in-vivo* in LRNC, fibrous tissue and recent IPH demonstrated the ability to discriminate plaque components on T_2_ maps.

## Background

Acute ischemic strokes are commonly associated with unstable carotid atherosclerotic plaques that can be detected *in-vivo* by multicontrast CMR [1-3]. Morphology and composition of atherosclerotic plaques can be characterized and classified following the CMR-modified American Heart Association (AHA) scheme [4]. The strength of multicontrast CMR resides in its ability to detect the presence of different plaque components by discriminating their relative signal intensities on time-of-flight (TOF), T_1_-, T_2_- and PD-weighted (T_1_W, T_2_W, PDW) images. Identification of lipid-rich necrotic core (LRNC), calcification, intraplaque haemorrhage (IPH) and fibrous tissue by *in-vivo* multicontrast CMR has been thoroughly validated by histology in several clinical studies [4-9].

The size of LRNC and the presence of IPH were shown histologically to be strongly associated with plaque instability [10]. Relative to the adjacent sternocleidomastoid muscle, MR signal from LRNC is iso/hyper-intense on TOF, T_1_W and PDW images, and hypo-intense on T_2_W images [7]. IPH is an independent predictor of future cardiovascular events [11] and reflects intraplaque neovascularization and plaque vulnerability [12]. It may also stimulate the progression of atherosclerosis by fuelling the plaque core with plasma membrane lipid from extravasated, broken-down, blood cells to increase the LRNC size [8]. MR signal from fresh IPH (type I) is hyper-intense on TOF and T_1_W images, and hypo/iso-intense on T_2_W and PDW images, whereas MR signal from recent IPH (type II) is iso/hyper-intense on all images [9].

Despite the advantages of multicontrast CMR, its non-quantitative nature and the need for extensive post-acquisition interpretation are barriers to the widespread clinical application of *in-vivo* CMR plaque characterization. Comparison of results from *in-vivo* CMR studies of atherosclerosis using different CMR systems and parameters can be challenging because plaque characterization is affected by variability and inconsistency in the signal intensity of plaque components relative to the reference intensity of the adjacent sternocleidomastoid muscle, which can be particularly evident in T_2_W images [2]. Furthermore, image intensity inhomogeneity caused by the surface coil sensitivity can be difficult to correct and can thus affect the relative signal intensities of plaque tissues and consequently the accuracy of plaque segmentation, in particular when automated methods are applied [13]. Finally, T_1_W and PDW images acquired with the Fast-Spin-Echo (FSE) sequence (used in multicontrast carotid CMR protocols) are known to suffer from blurring along the phase-encoding direction, which is inherent to the k-space acquisition strategy and causes a significant reduction of the vessel edge sharpness [14].

*In-vivo* quantitative mapping of relaxation times could provide an alternative method for plaque characterization. This technique measures the MR properties of plaque tissues directly, thus addressing the need for an absolute physical measure that can be standardized among different CMR systems and widely adopted in multi-centre studies, while at the same time avoiding the problems of image intensity inhomogeneity and blurring. Given these characteristics, quantitative mapping has great potential for automated plaque segmentation and classification and may provide new opportunities for quantification of plaque composition in carotid atherosclerosis that will be useful in clinical studies to evaluate emerging drugs that directly target plaque biology [15]. T_2_ relaxation times of LRNC and fibrous tissue found in the literature were all measured *ex-vivo*[16-23], except one study that compared *in-vivo* and *ex-vivo* T_2_ measurements [16]. To our knowledge, no *in-vivo* T_2_ measurements of IPH are reported in the literature.

The purpose of this study was thus to investigate the potential of quantitative T_2_ mapping as an *in-vivo* technique for atherosclerotic plaque characterization. In particular, we aimed to acquire *in-vivo* high-resolution quantitative T_2_ maps of carotid arteries, to study their use for AHA plaque type classification, and to measure *in-vivo* T_2_ relaxation times of the main plaque components at 3 T.

## Methods

### In-vivo CMR

The carotid arteries of 2 healthy volunteers and 15 patients with known atherosclerosis (11 males and 4 females, 71 ± 10 years, range 54-84) were imaged on two 3T scanners (12 patients on TIM Trio and 3 on Verio, Siemens Healthcare, both running VB17 software) using the Multiple-Spin-Echo (Multi-SE) sequence, called Spin-Echo-Multi-Contrast (SE_MC) on Siemens systems. Ethics approval from the local board was obtained and all subjects gave informed written consent. Black-blood cross-sectional 2D images of carotid arteries were acquired with the Machnet 4-channel phased-array carotid coil using Double-Inversion-Recovery (DIR) preparation and cardiac gating. Chemical shift selective fat saturation (FAT SAT) was used to suppress the signal from subcutaneous and perivascular fat, mainly composed of triglycerides, without affecting the signal from cholesterol and cholesteryl esters, the main lipids in atherosclerotic plaques [21,22]. If necessary, a saturation band was positioned on the anterior region of the neck to reduce ghosting artefacts from breathing and swallowing. Low Specific-Absorption-Rate (SAR) pulses were used in order to keep the total dissipated radio-frequency energy below the SAR limit without changing the flip angle of the refocusing pulses. Multi-SE parameters were TE = 12.9, 25.8, 38.7, 51.6, 64.5, 77.4, 90.3, 103.2 ms, TR = 2 R-R intervals, field of view (FOV) = 160 × 128 mm^2^ and matrix size = 320 × 256. Image resolution after zero-padding was 640 × 512 (pixel size = 0.25 mm). Slice thickness (2 mm) and pixel bandwidth (130 Hz/pix) were the same for Multi-SE and FSE. FSE T_1_W (TE = 14 ms, TR = 1 R-R and ETL = 9), T_2_W (TE = 89 ms, TR = 2 R-R and ETL = 15) and PDW (TE = 14 ms, TR = 2 R-R and ETL = 9) images had FOV = 150 × 150 mm^2^ and matrix size = 320 × 320. Image resolution after zero-padding was 640 × 640 (pixel size = 0.234 mm). TOF angiography was acquired using 3D Fast Low Angle Shot (FLASH) with flip angle = 18°, TR = 45 ms, TE = 4.1 ms, FOV = 200 × 150 mm^2^, matrix size = 256 × 192 and slice thickness = 1 mm. 69 TOF and 11 DIR-FSE T_1_W consecutive slices centred on the carotid bifurcation were first imaged in order to localize the atherosclerotic plaque. Then a series of DIR-FSE T_2_W and PDW (and if necessary other T_1_W) images were acquired to obtain full plaque coverage. Finally, a single-slice DIR-Multi-SE image series was acquired through the plaque centre. Given the constraints imposed by other imaging parameters, the TE series used in Multi-SE was defined considering an expected T_2_ ~ 50 ms for the normal carotid wall, which corresponded to a transverse magnetization half-life time T_2_·ln(2) ~ 35 ms. The last TE used in this study was approximately equal to 3 half-life times and typical SNR values measured on normal carotid wall went from ≥ 30 at the first echo to ≥ 4 at the last echo. Partial Fourier imaging was used to collect 5/8 of k-space (160 phase-encoding steps) and reduce the acquisition time of single-slice Multi-SE to circa 320 R-R intervals. The quality of Multi-SE and multicontrast images was assessed after each scan by two reviewers (A.C.L. and L.B.) who decided by consensus when carotid wall and plaque were not clearly visible.

### Pulse sequence

**Figure 1:**
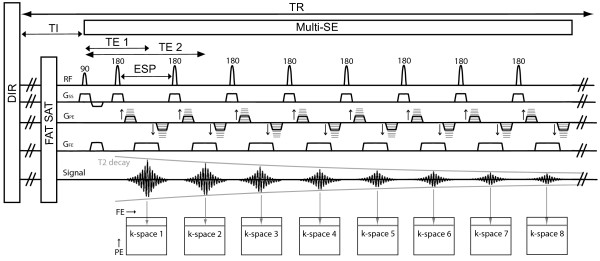
**Multi-SE sequence diagram with DIR and FAT SAT modules.** The RF excitation pulse is followed by a train of RF refocusing pulses. G_SS_ and G_FE_ are the slice selection and the frequency encoding gradients. The moment of the phase encoding gradient G_PE_ is the same along the pulse train and signal from consecutive echoes is encoded as lines of independent k-spaces. Phase encoding and rewinding gradients are applied before and after each signal acquisition window rather than a single phase encoding step applied before the first refocusing pulse. Constant crusher gradients at the left and right of the slice selective gradients are present in the sequence but are not shown in the diagram.

The Multi-SE sequence design is similar to the standard FSE that is widely used in carotid imaging, the main difference being the k-space acquisition strategy. Both are Carr-Purcell-Meiboom-Gill (CPMG) based sequences in which a 90° excitation pulse is followed by a train of 180° refocusing pulses to generate multiple spin echoes at every TR [24,25]. Constant crusher gradients at the left and right of each refocusing slice selective gradient are used to select both primary and stimulated echoes, which sum up at the mid-point between two consecutive refocusing pulses, whereas all other unwanted signal pathways are filtered out. In FSE each echo is phase-encoded to a different k-space line in order to collect multiple k-space lines for a single image during every TR, whereas in Multi-SE the phase-encoding is the same for the entire echo train. In this way, each echo of the Multi-SE sequence fills a line of a series of independent k-space planes, so that a set of images with different TEs are collected (Figure 1). Accuracy and precision improvements for clinical T_2_ mapping using a CPMG sequence have been demonstrated by setting the slice thickness of the refocusing pulses larger than that of the excitation pulse [26]. This simple modification stabilizes the flip angle of the refocusing pulse across each slice and minimizes refocusing profile errors. A 1.5 ratio between refocusing and excitation slice thickness was used in this study.

### T_2_ Mapping and plaque segmentation

Quantitative T_2_ maps of manually drawn regions of interest (ROIs) including vessels and muscles were generated from the Multi-SE series of images. The first image was discarded because of the different nature of the first echo with respect to the others. Since the flip angles of refocusing pulses are not exactly 180° due to B_1_ field inhomogeneities, only the signal acquired at the first TE is a pure primary echo, whereas signals acquired at the subsequent TEs are composed by primary and stimulated echoes. The signal intensity (SI) of the first echo will thus not fit in the exponential decay curve followed by the SIs of subsequent echoes, which are enhanced by stimulated echoes. For every ROI voxel the T_2_ mono-exponential decay curve *SI = β·e*^*-TE/T2*^ was therefore fitted to 7 SIs collected at different TEs (*β* includes the effect of proton density, T_1_ weighting, coil sensitivity, signal amplification and other factors).

**Figure 2:**
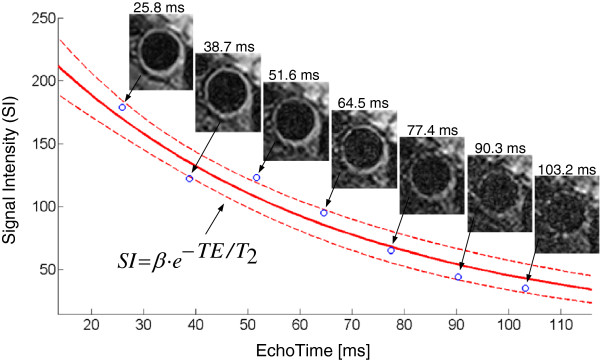
**Nonlinear fit of the T**_**2 **_**relaxation curve of a normal carotid artery.** The T_2_ mono-exponential decay curve (in red, with 95% confidence intervals) is fitted to the signal intensity (SI) of one voxel of the arterial wall in the Multi-SE image series. The voxel SI is represented by blue circles at 7 consecutive TEs.

The regression model used for T_2_ mapping consisted of a nonlinear monoexponential fit (Figure 2) initialized by T_2_ and *β* resulting from a robust linear fit of *ln(SI)*. Parameters estimated by linear least-squares regression are very sensitive to noise because the original error distribution becomes asymmetric after the logarithmic transform. To reduce this sensitivity to outliers, we used a robust regression method that iteratively minimizes the weighted sum of squares using bisquare weights, which depend on the residual of each data point [27]. To assess the statistical significance of fit parameter estimates, the two-tailed p-value of the ratio of each estimate to its standard error (derived from the diagonal elements of the estimated covariance matrix) was calculated on the t-distribution. Only linear fit parameter estimates rejecting the null hypothesis (p < 0.05) were used as initial values for the nonlinear fit in order to improve its convergence. Otherwise default initial values β = 500 and T_2_ = 50 ms were used. The nonlinear least-squares regression used the Levenberg-Marquardt algorithm [28]; data points at long TEs with SNR < 2 were discarded; 95% confidence intervals for the exponential curve (Figure 2) and for the fit parameters were estimated. Finally, only voxels with significant T_2_ and *β* estimates (p < 0.05) were accepted for generating T_2_ maps. The carotid arteries were subsequently segmented using a semi-automated method [29] that detected inner and outer wall boundaries on the second image (TE = 25.8 ms, TR = 2 R-R) of the Multi-SE series.

After this procedure, T_2_ maps of atherosclerotic arteries were segmented into 3 tissue types using a Bayes classifier [30] defined by the probability model *P(C*_*i*_*|T*_*2*_*) = P(C*_*i*_*)P(T*_*2*_*|C*_*i*_*)* combined with the maximum a posteriori (MAP) decision rule. *P(C*_*i*_*|T*_*2*_*)* with *i = 1,2,3* is the posterior conditional probability for a voxel with a specific T_2_ value to belong to one of 3 classes: LRNC, fibrous tissue or recent IPH. The MAP rule then assigned a particular voxel to the tissue type with the highest posterior probability. *P(C*_*i*_*|T*_*2*_*)* is determined by the prior probability *P(C*_*i*_*)*, which was assumed to be equal for every class, and the normal probability distribution *P(T*_*2*_*|C*_*i*_*)*, which was estimated from a set of approximately 100 voxels for each class. These voxels were manually selected from all the T_2_ maps to represent a specific tissue using multicontrast CMR as a guide, i.e. they were chosen inside a plaque component identified on multicontrast CMR [4,9]. In order to help matching the corresponding plaque features, multicontrast images were co-registered [31], so that inner and outer vessel boundaries detected on T_1_W images [29] could then be superimposed on PDW and T_2_W images. Calcification was identified by voxels inside the vessel wall boundaries with SNR < 2 at TE = 14 ms on the T_2_ decay curve (synthetic PDW image). Finally, mean ± SD of the T_2_ values of voxels classified as LRNC, fibrous tissue and recent IPH were calculated. The algorithms for quantitative T_2_ mapping, vessel wall segmentation and plaque classification were implemented in Matlab (Mathworks).

To compare atherosclerotic plaque characterization by T_2_ maps with conventional multicontrast CMR, two reviewers (A.C.L. and L.B.) independently classified the carotid arteries of 15 patients as normal or diseased (plaque type III, IV-V, VI, VII or VIII) following the CMR-modified AHA scheme [4,9]. One reviewer analysed multicontrast CMR (T_1_W, T_2_W and PDW image intensity inhomogeneity was corrected [13]) and TOF, while the other relied on T_2_ maps and TOF at matching slice locations. Both were blinded to the identification and the clinical data of each patient.

## Results

Out of 42 image series (including common, internal and external carotid arteries) acquired with single-slice Multi-SE in 15 patients, 5 were rejected due to poor image quality. The corresponding multicontrast (TOF, T_1_W, T_2_W and PDW) images were all accepted and the AHA plaque type classification performed on them by one reviewer found 14 normal and 23 diseased arteries (7 type III, 10 type IV-V, 2 type VI, 3 type VII and 1 type VIII plaques). AHA classification performed by the other reviewer on T_2_ maps and TOF found 11 normal and 26 diseased arteries (11 type III, 8 type IV-V, 3 type VI, 2 type VII and 2 type VIII plaques). The overall agreement on AHA plaque type between the reviewers was good (76% of matching classifications and Cohen’s κ = 0.68, Table 1).

**Table 1:** **AHA plaque type classification by multicontrast CMR vs. T**_**2 **_**maps + TOF**

**Multicontrast**	**T**_**2 **_**maps + TOF**						
**CMR**	**Normal**	**III**	**IV-V**	**VI**	**VII**	**VIII**	**Total**
Normal	10	4	…	…	…	…	14
III	1	6	…	…	…	…	7
IV-V	…	1	7	1	…	1	10
VI	…	…	…	2	…	…	2
VII	…	…	1	…	2	…	3
VIII	…	…	…	…	…	1	1
Total	11	11	8	3	2	2	37

**Figure 3:**
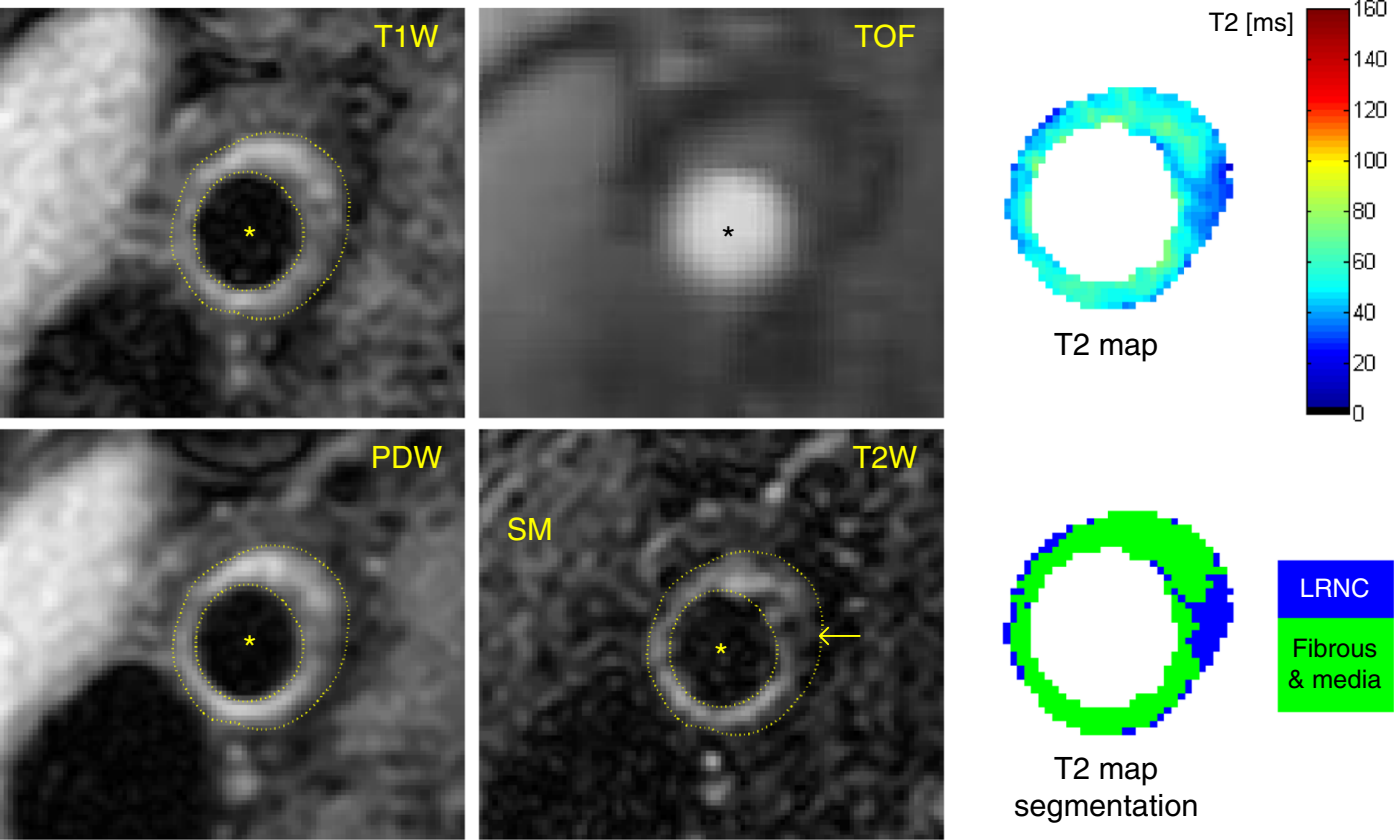
**Multicontrast CMR and T**_**2 **_**map of a type IV-V plaque in the right common carotid artery.** Multicontrast CMR shows a hypo-intense region compared to the sternocleidomastoid muscle (SM) on the T_2_W image, indicative of LRNC (arrow). The T_2_ map and its segmentation show a thick fibrous cap separating the LRNC, characterized by shorter T_2_ values, from the lumen.

**Figure 4:**
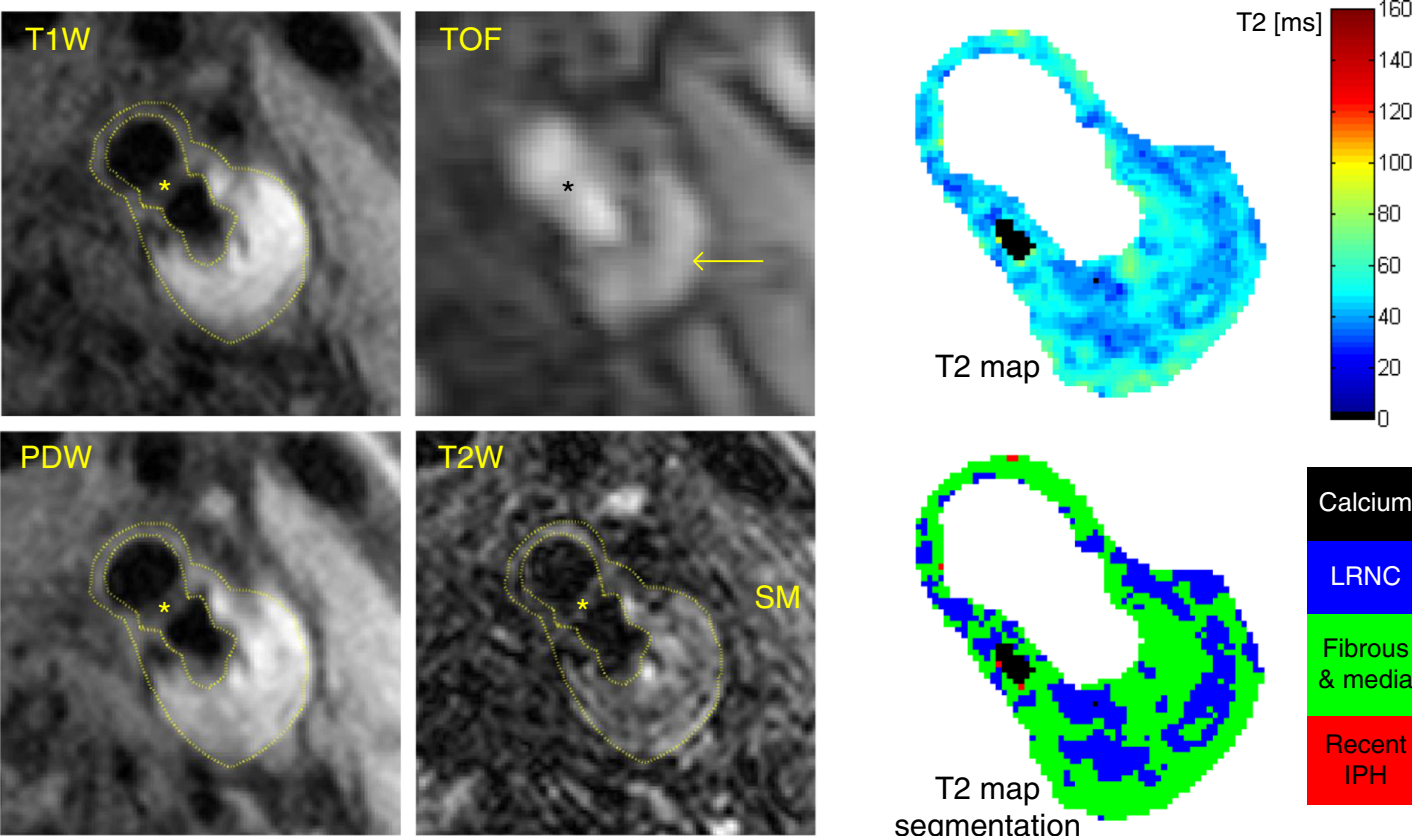
**Multicontrast CMR and T**_**2 **_**map of a large type VI plaque at the left carotid bifurcation.** Multicontrast CMR reveals the presence of fresh IPH (arrow), characterized by hyper-intensity on TOF and T_1_W images compared to the sternocleidomastoid muscle (SM). The T_2_ map and its segmentation illustrate that the plaque is composed by fibrous tissue, pools of lipid and necrotic material (LRNC) and a small calcification. Some sparse voxels were classified as recent IPH due to T_2_ overestimation.

The T_2_ maps of the 10 arteries classified as normal by both reviewers, together with 4 common carotid arteries from 2 healthy volunteers, were used to measure T_2_ mean ± SD of normal carotid wall (T_2_ = 54 ± 13 ms, Table 2). The T_2_ maps of the 15 atherosclerotic arteries with advanced plaques (graded as type IV-V, VI, VII or VIII by both reviewers) were segmented by the Bayes classifier into 4 plaque components: calcification, LRNC, fibrous tissue and recent IPH. From a total of 11865 voxels, the T_2_ measurements of 668 voxels were rejected because not statistically significant (p > 0.05). 192 voxels were identified as calcification in one type IV-V, one VI and two VII plaques. 2394 voxels with T_2_ = 37 ± 5 ms were classified as LRNC by the segmentation algorithm, mostly detected in type IV-V and VI plaques (Table 2). 7511 voxels with T_2_ = 56 ± 9 ms were classified as fibrous tissue and normal intima/media (Table 2). Figure 3 shows an atherosclerotic artery with LRNC and a thick fibrous cap separating it from the lumen (type IV-V). Figure 4 shows a large complex plaque (type VI) at the carotid bifurcation composed of lipid and necrotic material mixed together with fibrous tissue. These features agreed with the multicontrast CMR classification, which revealed the presence of fresh IPH (hyper-intensity on TOF and T_1_W images).

**Table 2:** **T**_**2 **_**measurements (mean ± SD) of arterial wall and plaque tissues**

**Study**	**Field strength**	**Num. of TEs**	**Num. of plaques**	**Location**	**LRNC [ms]**	**Fibrous tissue [ms]**	**Intima & media [ms]**	**Ref.**
**in-vivo**	**3 T**	**7**	**15**	**carotid**	**37 ± 5**	**56 ± 9**	**54 ± 13**	*This study
**in-vivo**	**1.5 T**	**2**	**7**	**carotid**	**28 ± 6**	**51 ± 10**	**48 ± 7**	[16]
ex-vivo	1.5 T	2	7	carotid	31 ± 5	51 ± 9	52 ± 7	[16]
ex-vivo	1.5 T	8	10	carotid	47 ± 14	60 ± 13	67 ± 23	[17]
ex-vivo	1.5 T	4	10	carotid	84 ± 41	95 ± 32	60 ± 6	[18]
ex-vivo	9.4 T	7	3	carotid	35-49	48-60	72-76	[19]
ex-vivo	4.7 T	4	7	coronary	31 ± 7	55 ± 11	50 ± 10	[20]
ex-vivo	1.5 T	10	8	various	55 ± 3	79 ± 4	81 ± 3	[21]
ex-vivo	4.7 T	10	8	various	50 ± 3	63 ± 1	65 ± 2	[21]
ex-vivo	9.4 T	20	8	various	20 ± 3	30 ± 2	30 ± 3	[21]
ex-vivo	1.5 T	32	13	various	< 20	100-200	68 ± 10	[22]
ex-vivo	3 T	7	14	aorta/iliac	54 ± 3	89 ± 6	76 ± 9	[23]

*Fibrous tissue and normal arterial wall cannot be discriminated *in-vivo* by T_2_ alone. This study measured the T_2_ values of tunica intima and media in 14 normal arteries, while both fibrous tissue and normal arterial wall of the 15 atherosclerotic arteries were categorized together as fibrous tissue.

**Figure 5:**
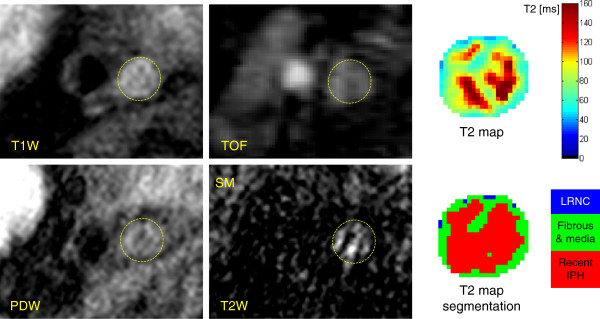
**Multicontrast CMR and T**_**2 **_**map of an occlusive type VI plaque in the right internal carotid artery.** Multicontrast CMR reveals the presence of recent IPH, characterized by iso/hyper-intensity on TOF, T_1_W and T_2_W images compared to the sternocleidomastoid muscle (SM). The T_2_ map and its segmentation show a large region with long T_2_ values, corresponding to recent IPH, surrounded by fibrous tissue. Due to the borderline quality of multicontrast CMR, it was not possible to co-register the images and segment the vessel wall, therefore a circle was manually placed to indicate the occluded artery.

Finally, large areas of recent IPH were detected on T_2_ maps in two type VI plaques for a total of 528 voxels measuring T_2_ = 105 ± 25 ms. However, only the plaque with recent IPH in Figure 5 (236 voxels with T_2_ = 107 ± 25 ms) was confirmed as type VI by multicontrast CMR. In the other 13 plaques, sparse voxels and small regions (for a total of 572 voxels with T_2_ = 86 ± 9 ms) were classified as recent IPH by the segmentation algorithm. Additionally, since most clinical CMR studies of atherosclerosis used the sternocleidomastoid muscle as a signal intensity reference for plaque tissue classification in T_2_W images [4,7,9], we generated T_2_ maps of sternocleidomastoid muscles in volunteers and patients and measured T_2_ = 39 ± 6 ms.

## Discussion

The objective of this study was to investigate the use of T_2_ mapping for plaque characterization. We acquired *in-vivo* high-resolution T_2_ maps of carotid arteries in 15 atherosclerotic patients using the Multi-SE sequence and demonstrated good agreement between AHA plaque type classified by T_2_ maps and TOF images and by conventional multicontrast CMR. The present study measured the T_2_ of LRNC, fibrous tissue and recent IPH and showed the potential of T_2_ mapping for plaque segmentation and classification. Our *in-vivo* T_2_ measurements (Table 2) were slightly longer but broadly in agreement with the only *in-vivo* T_2_ study of carotid atherosclerosis available in the literature [16]. The T_2_ values of tunica intima and media measured in 14 normal carotid arteries fell within the T_2_ range reported by *ex-vivo* studies of carotid and coronary atherosclerosis [16-20]. With the exception of one study that reported much longer values [18], the T_2_ measurements of fibrous tissue and LRNC in 15 advanced plaques were comparable with those obtained *ex-vivo*[16,17,19,20]. Recent IPH was characterized by long T_2_, consistent with signal hyper-intensity on T_2_W images [9], and was detected in one complex (type VI) plaque (confirmed by multicontrast CMR). In other plaques the classification of some sparse voxels and small regions as recent IPH was most likely caused by T_2_ overestimation errors: their T_2_ values were longer than their neighbouring voxels but shorter than the complex plaque and TOF images showed no signs of IPH. The measured T_2_ of sternocleidomastoid muscles agreed with the range of values reported in literature for muscle tissue (30-50 ms) at 1.5 T and 3 T [32]. It is slightly longer than the T_2_ of LRNC but shorter than that of normal carotid wall and fibrous tissue, confirming it as a suitable intensity reference in T_2_W images [4,7,9]. However, intensity inhomogeneity introduced by the sensitivity profile of surface coils, which strongly affects the sternocleidomastoid muscle due to its skin proximity, must be corrected before it can be used as reference.

Table 2 shows the *ex-vivo* studies of atherosclerosis that were found in literature [16-23]. Despite their methodological differences (i.e. field strength, imaging parameters and arterial locations from which the plaques had been excised), the common feature of these *ex-vivo* studies is a shorter T_2_ in LRNC than in fibrous tissue and normal intima and media, which was confirmed by our *in-vivo* T_2_ measurements. However, care must be taken when comparing *in-vivo* and *ex-vivo* T_2_ values, because the effect of temperature and fixatives on tissue specimens can be significant [18]. Five *ex-vivo* CMR studies were performed at body temperature [16,18,20,21,23] and three at room temperature [17,19,22].

Our *in-vivo* T_2_ measurement method is more accurate than the previous *in-vivo* study [16], which calculated T_2_ of 7 carotid arteries by collecting and fitting only 2 echoes with relatively short TE (20 and 55 ms). In that study [16] the high sensitivity to noise due to the limited number of echoes constraining the T_2_ exponential decay curve was aggravated by the use of short TR (1 R-R),which causes more T_1_ weighting, and very thick slices (5 mm), which can mix T_2_ values of different tissues (partial volume averaging). We used a CPMG sequence (Multi-SE) to acquire more echoes (7 TEs ranging from 25.8 to 103.2 ms) designed to obtain the best possible sampling of the T_2_ relaxation decay curve of target tissues. The Multi-SE acquisition parameters were more appropriate for T_2_ quantitation than those used by the previous *in-vivo* study, since TR was longer (2 R-R) and slices thinner (2 mm). We also implemented a nonlinear regression method that is more robust to outliers, and we ensured that the T_2_ estimates were statistically significant by accepting only results with p < 0.05. Finally, the present study imaged 14 normal and 15 atherosclerotic carotid arteries, more than those analysed previously (Table 2).

In some cases the first image of the Multi-SE series showed artefacts due to residual signal from slowly flowing blood not suppressed by DIR. We speculated that the main cause was inaccurate shimming of B_0_ inhomogeneities. Flow artefacts did not produce T_2_ errors because the first contrast image was never used to estimate T_2_, as explained in the methods.

### Limitations

The main limitation of this study is the absence of plaque histology, since only two of the participating patients underwent surgery and their endarterectomy specimens were of insufficient quality for detailed analysis. However, plaque characterization performed on *in-vivo* T_2_ maps agreed with results of multicontrast CMR, which has been previously validated by histology [4-9]. A physical limitation of T_2_ mapping is that the presence of fresh IPH (type I) cannot be detected by T_2_ alone. Fresh IPH infiltrating the LRNC is characterized by short T_2_[8,9], so it can only be detected using T_1_ information, e.g. independent identification on TOF images (Figure 4).

Finally, imperfections of the refocusing pulses represent the main source of errors in T_2_ quantitation [33], although the CPMG sequence used in this study partially compensates for them. Stimulated echoes produced by non-ideal refocusing pulses are acquired together with the primary echoes, thus introducing T_1_ weighting into the signal and altering the pure T_2_ relaxation decay. After discarding the first echo, which is purely primary, the contribution of stimulated echoes to the remaining 7 echoes may have caused T_2_ overestimation. These errors depend on the amount of T_1_ weighting introduced by the use of TR = 2 R-R. A very long TR (~5·T_1_ of target tissue) would limit the T_1_ effect, but it would be absolutely impractical under *in-vivo* high-resolution imaging conditions. Finally, the elimination of the first echo may have affected the estimation of short T_2_ components in the LRNC (~35 ms), which have a transverse magnetization half-lifetime (~24 ms) as long as the second TE. In general, this problem can be alleviated by the use of shorter echo spacing (ESP). This study used the shortest ESP possible, given the requirements for matrix size, receiver bandwidth and the duration of low-SAR refocusing pulses. Future developments will aim at improving *in-vivo* T_2_ measurement accuracy, decreasing acquisition time and validating plaque characterization with histology.

## Conclusions

This study draws attention to the potential use of *in-vivo* quantitative T_2_ mapping for carotid plaque characterization. On 37 carotid arteries imaged *in-vivo* in 15 atherosclerotic patients, AHA plaque type classification using high-resolution T_2_ maps and TOF images showed good agreement with conventional multicontrast CMR. T_2_ relaxation times measured *in-vivo* at 3 T for tunica intima and media in 14 normal arteries and for LRNC, fibrous tissue and recent IPH in 15 arteries with advanced plaques demonstrated the ability to discriminate 4 major plaque components including calcification, thus supporting the application of plaque segmentation and classification based on T_2_ maps.

## Competing interests

The authors declare that they have no competing interests.

## Authors’ contributions

LB, RPC and MDR provided the concepts. LB, ACL and MDR developed the CMR protocol. ACL and JTC recruited the patients. LB, ACL, JTC and MDR collected data. LB and ACL reviewed data. LB developed algorithms, analysed data and drafted the manuscript. RPC and MDR supervised the project and acted in last author capacity. All authors read and revised the manuscript critically for important intellectual content, and approved the final version.
